# Dysregulated Inflammatory Cytokine Levels May Be Useful Markers in a Better Up-Dated Management of COVID-19

**DOI:** 10.3390/cimb46080525

**Published:** 2024-08-15

**Authors:** Marco Iuliano, Roberta Maria Mongiovì, Alberico Parente, Blerta Kertusha, Anna Carraro, Raffaella Marocco, Giulia Mancarella, Cosmo Del Borgo, Laura Fondaco, Lorenzo Grimaldi, Maria Dorrucci, Miriam Lichtner, Giorgio Mangino, Giovanna Romeo

**Affiliations:** 1 Department of Medico-Surgical Sciences and Biotechnologies, Sapienza University of Rome, 04100 Latina, Italy; marco.iuliano@uniroma1.it (M.I.); giorgio.mangino@uniroma1.it (G.M.); 2 Department of Biomedical and Dental Sciences and Morphofunctional Imaging, University of Messina, 98122 Messina, Italy; robertamaria.mongiovi@gmail.com; 3 Department of Public Health and Infectious Disease, S. Maria Goretti Hospital, Sapienza University of Rome, 04100 Latina, Italy; alberico.parente@uniroma1.it (A.P.); blerta.kertusha@uniroma1.it (B.K.); anna.carraro@uniroma1.it (A.C.); raffaella.marocco@uniroma1.it (R.M.); giulia.mancarella@uniroma1.it (G.M.); cosmo.delborgo@uniroma1.it (C.D.B.); laura.fondaco@uniroma1.it (L.F.); 4 Department of Infectious Diseases, Istituto Superiore di Sanità, 00161 Rome, Italy; maria.dorrucci@iss.it; 5 Department of General Surgery and Surgical Specialty, Sapienza University of Rome, 00168 Rome, Italy; miriam.lichtner@uniroma1.it

**Keywords:** SARS-CoV-2, inflammatory mediators, IL-10, IP-10

## Abstract

Coronavirus disease 2019 (COVID-19) is an infection characterized by the dysregulation of systemic cytokine levels. The measurement of serum levels of inflammatory cyto-/chemokines has been suggested as a tool in the management of COVID-19. The aim of this study is to highlight the significance of measured levels of interleukin (IL)-1α, IL-1β, IL-6, IL-8, IL-10, IL-12(p70), IL-27, interferon (IFN)γ, interferon gamma-induced protein (IP)-10, monocyte chemoattractant protein (MCP)-1, and tumor necrosis factor (TNF)-α in serum samples from infected and recovered subjects, possibly predictive of severity and/or duration of the disease. Serum samples from healthy (HD), positive at hospital admittance (T0), and recovered subjects (T1, 31–60, or 70–200 days post-negativization) were collected and tested through a bead-based cytometric assay and confirmed through ELISA. IL-10 levels were increased in the T0 group compared to both HD and T1. IL-27 significantly decreased in the 31–60 group. IL-1β significantly increased in the 70–200 day group. TNF-α significantly decreased in T0 compared to HD and in the 31–60 group versus HD. IP-10 significantly increased in T0 compared to HD. These results suggest that IP-10 could represent an early marker of clinical worsening, whereas IL-10 might be indicative of the possible onset of post-COVID-19 long syndrome.

## 1. Introduction

Coronavirus disease 2019 (COVID-19) is caused by a respiratory tract infection with Severe Acute Respiratory Syndrome Coronavirus-2 (SARS-CoV-2), a virus belonging to the beta genus of the *Coronaviridae* family. Infection of the lower respiratory tract results in pneumonia, then leading, in the most serious prognoses, to death [1]. SARS-CoV-2 infection has evolved alongside the evolution of the virus by changing the speed of infection, transmission, and the type of symptoms [2]. Following the massive vaccination program, the percentage of serious recurrences of the disease has drastically diminished, although the spread of the virus has yet to be blocked [3]. This is principally due to the inability of the vaccines to induce a sterilizing immunity [4].

It is known that all of the variants of SARS-CoV-2 share the ability to specifically alter the interleukin release (i.e., IL-6, IL-10, IL-18, and IL-27) and to induce a strong inflammatory response that possibly leads to a so-called “cytokine storm”, i.e., an excessive and prolonged secretion of inflammatory cytokines that leads to multiorgan damage [5]. Most of the research focused attention on the first COVID-19 wave, in which high concentrations of IL-1β, tumor necrosis factor (TNF)-α, IL-6, IL-10, and interferon γ-induced protein (IP)-10 were observed in patients with severe forms of COVID-19. These circumstances are often indicative of a fatal outcome of the disease. Moreover, results regarding cytokine levels that are reported in the literature are often contrasting, due to several factors: time of blood withdrawal (i.e., at admission, during the acute phase, at the resolution, and in the convalescence phase), interindividual variables (i.e., age, gender, comorbidities, etc.) and genetic background (i.e., mutations or genetic polymorphisms in the genes encoding for key elements of the innate and/or adaptive immune responses) [6,7].

While the analysis of cyto-/chemokine serum levels has been widely performed on COVID-19 seropositive patients, the alteration in the expression pattern of inflammatory cytokines in convalescent subjects has been less investigated. Indeed, convalescent subjects were mainly tested for the presence or absence of viral antigens and specific antibodies against them in order to observe the spreading of the pandemic and to control the infection progression. Nevertheless, the chronic, low-grade activation of the innate immune response leading to the dysregulation of cyto-/chemokine production in convalescent subjects could lay the foundations to some of the symptoms typically associated with long COVID-19 syndrome such as the following: fatigue, shortness of breath, cough, muscle, joint and chest pain, depression, headaches, rapid heartbeat, and fever [8].

The rapid evolution of COVID-19 was contrasted with the efficient procedure to individuate and manage such a disease. The study of classical inflammatory mediators of the host could represent a valid system to individuate a constant trace alongside the virus evolution. Moreover, it is reasonable to think that only a specific combination of cyto-/chemokine alteration could effectively be predictive for the disease evolution.

By testing the serum levels of some non-viral immune markers connected to COVID-19, like inflammatory cyto-/chemokines, the aim of this work is to improve our understanding of these biomarkers as factors for an updated clinical management of COVID-19. We screened COVID-19 patients in both acute and convalescent phases by using a multiplex cyto-/chemokine bead-based cytometric assay comparing COVID-19 patients and convalescent and healthy subjects.

## 2. Materials and Methods

### 2.1. Study and Population Design

A transversal analysis was performed comparing patients with COVID-19 at the time of acute onset at hospital admission (PZ T0 *n* = 31) and outgoing patients followed at a post-COVID clinic with a median time of follow-up of 32 ± 39.15 (range 2–207 days) (PZ T1 *n* = 39). All the subjects were Caucasian and came from the province of Latina and Rome. The study was conducted from March 2020 to the beginning of 2022.

A group of healthy donors (HD *n* = 25), administered with at least one dose of vaccine, who never experienced COVID-19, was used as control. The last dose of vaccine was administered to HD at least 2 months before the blood withdrawal used for cyto-/chemokine assessment. Patients were further grouped according to disease severity expressed by *p*/F value. ‘Mild’ severity was defined when the arterial blood oxygen partial pressure (PaO2)/oxygen concentration (FiO2, *p*/F value) exhibited levels between 200 and 300 mmHg. The ‘Severe’ state of the disease was defined with a *p*/F value below 200 mmHg with recommended patient intubation and admission to an Intensive Care Unit. A 5 mL blood withdrawal was taken from each participant; serum was separated by centrifugation for 10 min at 2000× *g* and 4 °C and immediately stored at −80 °C until measurement. The cohort of subjects for ELISA analysis was expanded by including 15 new healthy donors and 11 new patients. The patients were further divided according to the elapsed time since the end of the infection: 2–30 days, 31–60 days, and 70–207 days. Clinical characteristics at baseline and in the follow-up were collected. See Table 1 and Table 2 for detailed information.

### 2.2. Multiplex Cytokine Assay

Collected supernatants were thawed on ice and analyzed using the “Super-X Plex Cytokine Assay” (Antigenix America, Huntington, NY, USA) for the detection of 11 inflammatory cyto-/chemokines (i.e., IL-1α, IL-1β, IL-6, IL-8, IL-10, IL-12(p70), IL-27, IFNγ, IP-10, MCP-1, and TNFα) according to the manufacturer’s instructions. All the data were acquired on a FACs ARIA II Sorter (Becton Dickinson, Franklin Lakes, NJ, USA) using FACs DiVA software (v6.3.1). Cytometric analysis was performed according to the manufacturer’s instructions and at least 100 events for each bead population in the gate corresponding to larger beads were acquired. Data were analyzed using Flowing Software (v2.5.1, University of Turku, Turku, Finland). Threefold serial dilutions of the standard solution were used to extrapolate the concentration of each cytokine from the Mean Fluorescence Intensity (MFI) of the phycoerythrin signal for each bead population.

### 2.3. Human Cytokines Sandwich ELISA Array

Cytokines were analyzed using the following Human Cytokine Sandwich ELISA kits: human TNF-α (sensitivity: 9.375 pg/mL), IL-27 (sensitivity: 94 pg/mL), and IP-10 (sensitivity: 4.688 pg/mL) were purchased from Fine test (Wuhan, China); ELISA kits for human IL-1β (sensitivity: 0.4 pg/mL) and IL-10 (sensitivity: 1 pg/mL) were purchased from Mabtech (Nacka Strand, SE, Sweden); and human IFN-α (sensitivity: 1.95 pg/mL) was purchased from PBL Assay Science (Piscataway, NJ, USA). According to manufacturers’ indications, each kit was pre-tested on 8 samples to evaluate the average concentrations of every specific cytokine, thereby allowing the choice of the appropriate dilution for optimal cytokine detection and setting the duration of 3,3′,5,5′-Tetramethylbenzidine (TMB) incubation. All the other steps were performed according to manufacturers’ instructions for each ELISA kit. All the samples were measured in duplicate.

At the end of the procedure, the microplates were read at a 450 nm wavelength using a Sunrise absorbance reader (Tecan Magellan, Männedorf, Switzerland). Obtained data were collected and analyzed by using Excel (Microsoft office, Redmond, WA, USA) version 16.43.

### 2.4. Principal Component Analysis (PCA)

Principal component analysis was performed by using the “PCA method” of the R package (https://biit.cs.ut.ee/clustvis/#editions; accessed on 12 July 2024) on the ClustVis website [9]. The analysis was conducted on the concentration values (expressed in pg/mL) of the six cytokines (IL-10, IP-10, TNF-α, IFN-α IL-1β, and IL-27) obtained by ELISA assay for all the analyzed samples.

### 2.5. Statistical Analysis

A two-tail *p*-value Mann–Whitney *t*-test was performed to compare two sample groups. To compare three or more groups, non-parametric ANOVA with Dunnett’s multiple comparisons test was performed. Here, *: *p* < 0.05; **: *p* < 0.01; and ***: *p* < 0.001. All data were presented as mean ± SD. Graphs were produced using GraphPad Prism version 8 software (Dotmatics, San Diego, CA, USA) and ClustVis algorithm website (https://biit.cs.ut.ee/clustvis/; accessed on 12 July 2024). To evaluate the association between cytokine concentration and severity of COVID-19, we applied separate univariate logistic regression models at T0 and at T1 as described: at T0 severe COVID-19 (vs. mild) was the outcome whilst high cytokine (vs. low) was the possible predictor, while at T1, during convalescence, high cytokine concentration (vs. low) or low cytokine concentration (vs. high) was the event of interest, based on an Odds Ratio (OR) > 1; in this last case, severe COVID-19 (vs. mild) was the predictor variable. In order to determine high and low cytokine concentration, we considered the quintiles of cytokine distributions at T1 and we created five dichotomous variables according to the quintiles, assuming as “high” the values of cytokines ≥ each quintile and as “low” the values < each considered quintile; finally, we chose, as the cut-point of the cytokines, the quintile for which the OR was statistically significant > 1 for severe COVID-19 (vs. mild). When we found a statistically significant association (i.e., OR > 1 with *p* < 0.05) at T1 we applied bivariate logistic models to estimate the OR of high vs. low cytokines adjusted for elapsed time and for natural log-transformed cytokine concentration at T0.

### 2.6. Ethics

The Research Ethics Committee “Lazio 2” of Sapienza University of Rome evaluated and approved this research, with the assigned protocol number 0038491, 17 February 2022.

## 3. Result

### 3.1. Concentrations of IL-10, IP-10, TNF-α, IL-1β, IL-27, and IL-8 Are Deregulated in Serum from Patients with COVID-19

**Figure 1:**
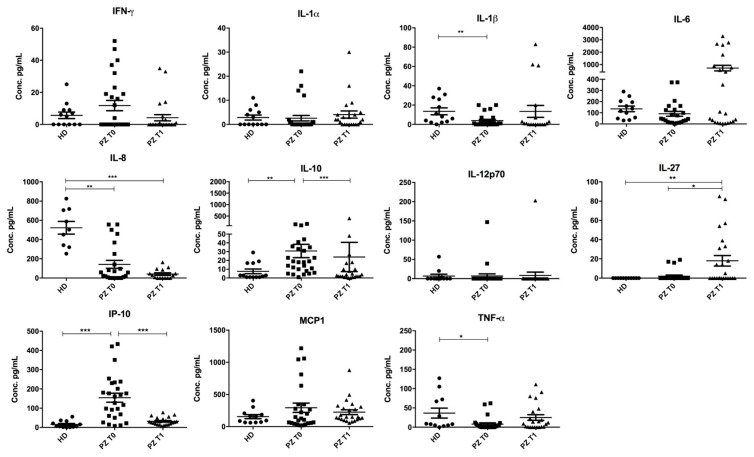
Cyto-/chemokine serum expression levels in patients analyzed by the “Super-X Plex Cytokine Assay” (Antigenix America, Huntington, NY, USA) during COVID-19 disease (PZ T0), after 40–170 days from hospital dismission (PZ T1), and in the control group that includes healthy subjects vaccinated with at least one dose of vaccine (HD). All data were presented as mean ± SD and values obtained by statistical analysis of variance ANOVA. Significant differences were represented with *: *p* < 0.05; **: *p* < 0.01; and ***: *p* < 0.001.

Firstly, an overview of the cytokine and chemokine landscape of the serum of COVID-19-affected (PZ T0) and recovered subjects (PZ T1) was performed on a series of representative samples in order to define the best cytokine and chemokine combination able to describe the inflammatory scenario. To accomplish such a task, we performed the quantification of serum inflammatory cyto-/chemokine concentrations by implementing a multiplex cytometric bead-based assay. While no significant differences emerged for IL-6, IL-1α, IFN-γ, MCP1, and IL-12p70, the levels of IL-1β, IL-8, IL-10, IL-27, IP-10, and TNF-α appeared deregulated in serum from patients with COVID-19 compared to healthy donors (HD) (Figure 1). Specifically, we observed an increase in both IL-10 and IP-10 in the PZ T0 group compared to HD controls (IL-10 PZ T0: 31.77 pg/mL ± 39.90 pg/mL vs. IL-10 HD: 5.75 pg/mL ± 7.25 pg/mL, *p* < 0.01; IP-10 PZ T0: 154.50 pg/mL ± 119.28 pg/mL vs. IP-10 HD: 15.50 pg/mL ± 17.89 pg/mL, *p* < 0.001) followed by a decrease from the PZ T0 to the PZ T1 group (IL-10 PZ T0: 31.77 pg/mL ± 39.90 pg/mL vs. IL-10 PZ T1: 24.83 pg/mL ± 83.18 pg/mL, *p* < 0.001; IP-10 PZ T0: 154.50 pg/mL ± 119.28 pg/mL vs. IP-10 PZ T1: 31.91 pg/mL ± 19.55 pg/mL, *p* < 0.001). TNF-α and IL-1β significantly decreased in the PZ T0 group compared to HD (PZ T0: 8.08 pg/mL ± 16.90 pg/mL vs. HD: 36.50 pg/mL ± 44.67 pg/mL, *p* < 0.05 and PZ T0: 3.88 pg/mL ± 6.71 pg/mL vs. HD: 13.54 pg/mL ± 12.86 pg/mL, *p* < 0.01, respectively) while no differences were observed in the PZ T1 group. IL-27 was significantly increased in PZ T1 compared to HD as well as in PZ T1 compared to PZ T0 (PZ T1: 18.00 pg/mL ± 26.91 pg/mL vs. HD: 0 ± 0, *p* < 0.01 and vs. PZ T0: 2.08 pg/mL ± 5.77 pg/mL, *p* < 0.05, respectively). IL-8 significantly decreased in PZ T0 compared to HD as well as in PZ T1 compared to the HD group (HD: 522.44 pg/mL ± 199.81 pg/mL vs. PZ T0: 142.48 pg/mL ± 197.78 pg/mL *p* < 0.01 and vs. PZ T1: 41.00 pg/mL ± 51.04 pg/mL, *p* < 0.001, respectively).

### 3.2. IL-1β, IL-10, IL-27, IP-10, and TNF-α Dysregulations Are Confirmed by ELISA Assay in an Extended Cohort

Based on the reported results, we focused our attention on IL-10, IP-10, TNF-α, IL-1β, and IL-27. We, therefore, proceeded to quantify these selected cytokines by a sandwich ELISA. Moreover, an IFN-α dosage (not present in the cytometric bead-based assay previously used) was added due to the central role of this type of cytokine during viral infection.

We extended our screening to 58 subjects that were divided into five groups depending on the elapsed time from the end of the SARS-CoV-2 infection: HD, PZ T0, recovered subjects from 2 to 30 (PZ T1_2–30), 31 to 60 (PZ T1_31–60), and from 70 to 207 days (PZ T1_70–207).

**Figure 2:**
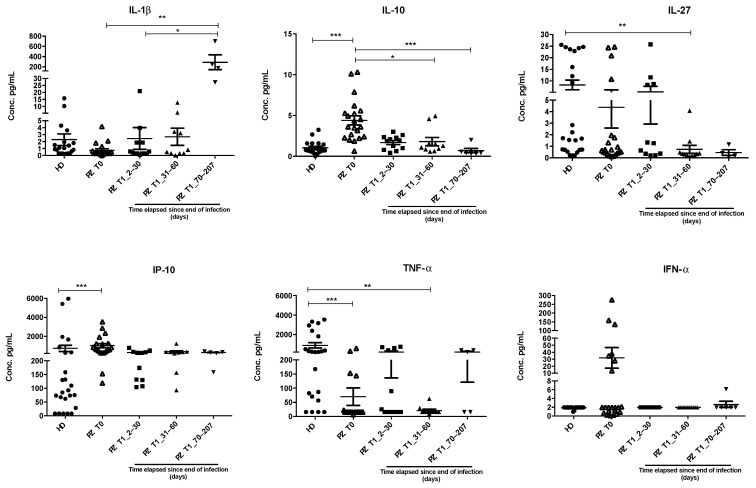
Scatter plots representing the dosages of the cyto-/chemokines IL-10, IL-27, IL-1β, IFN-α, TNF-α, and IP-10 into the serum of 58 subjects analyzed using a Human Cytokine Sandwich ELISA array kit. The subjects were clustered in 5 groups: HD, which includes healthy subjects with at least one dose of vaccine; positive group (PZ T0), affected individuals whose serum was collected at the time of admission; 2–30, 31–60, and 70–207 day groups that include recovered patients clustered based on the time elapsed from the end of the infection (PZ T1_2–30, PZ T1_31–60, PZ T1_70–207, respectively). All data were presented as mean ± SD and values obtained by statistical analysis of variance ANOVA. Significant differences were represented with *: *p* < 0.05; **: *p* < 0.01; and ***: *p* < 0.001.

Following the dosage of these cyto-/chemokines (Figure 2), we found that IL-1β seemed to maintain rather low concentrations in all the examined groups, significantly increasing in the 70–207 day group (PZ T1_70–207: 289.08 pg/mL ± 290.58 pg/mL vs. PZ T0: 0.73 pg/mL ± 1.00 pg/mL, *p* < 0.01 vs. PZ T1_2–30: 2.45 pg/mL ± 5.66 pg/mL, *p* < 0.05, respectively). IL-10 was statistically increased in the positive group compared to the HD group, as well as compared to the groups 31–60 and 70–207 days from the end of the infection (PZ T0: 4.40 pg/mL ± 2.57 pg/mL vs. HD: 1.06 pg/mL ± 0.77 pg/mL, *p* < 0.001, vs. PZ T1_31–60: 1.80 pg/mL ± 1.60 pg/mL, *p* < 0.001 and vs. PZT1_70–207: 0.69 pg/mL ± 0.65 pg/mL, *p* < 0.05). IL-27 showed higher concentrations in healthy subjects, which significantly decreased in the 31–60 day group (HD: 8.62 pg/mL ± 10.30 pg/mL vs. PZT1_31–60: 0.74 pg/mL ± 1.16 pg/mL, *p* < 0.01). IP-10 significantly increased in the positive group compared to the control (PZ T0: 1022.06 pg/mL ± 946.40 pg/mL vs. HD: 762.42 pg/mL ± 1641.71 pg/mL, *p* < 0.001). TNF-α significantly decreased in the positive group as well as in the 31–60 day group compared to control (PZ T0: 69.99 pg/mL ± 141.77 pg/mL vs. HD: 905.18 pg/mL ± 1276.56 pg/mL, *p* < 0.001 and PZ T1_31–60 days: 21.05 pg/mL ± 15.31 pg/mL vs. HD: 905.18 pg/mL ± 1276.56 pg/mL, *p* < 0.01). Finally, even if IFN-α seems more abundant in the sera of positive patients compared to the levels measured in all the other groups, this difference was not statistically significant (*p* = 0.95).

### 3.3. IP-10 and TNF-α Dysregulations Are Associated with COVID Severity

**Figure 3:**
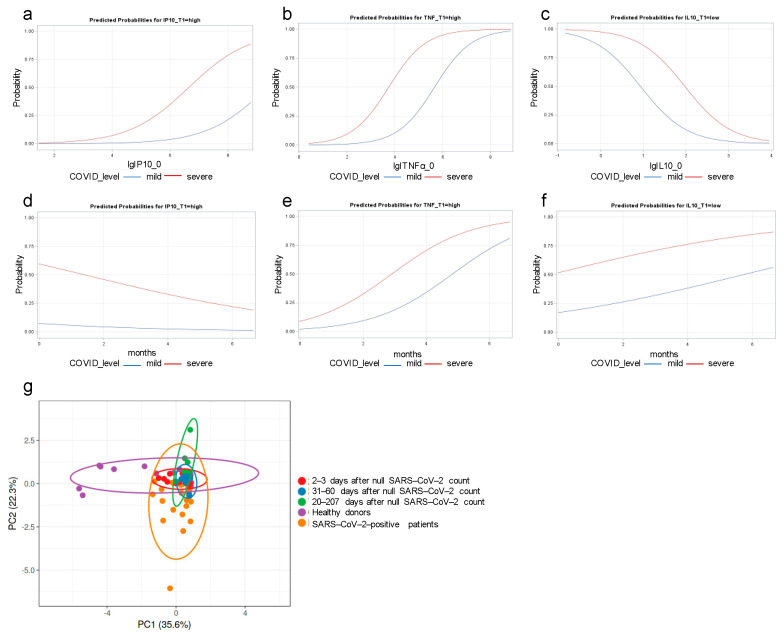
The association between clinicopathological factors and cytokine concentrations of COVID-19 patients was evaluated in separate univariate logistic regression models as predicted probabilities for IP-10 (**a**), TNF-α (**b**), and IL-10 (**c**) and by controlling the time elapsed from negative test for IP-10 (**d**), TNF-α (**e**), and IL-10 (**f**), respectively. Principal Component Analysis (PCA) (**g**) was performed on the concentration values expressed in pg/mL of the cytokines IL-10, IP-10, TNF-α, IFN-α, IL-1β, and IL-27 obtained by ELISA assay. Each point represents the variance of each of the 80 samples analyzed and clustered in 5 groups: Ctrl, Pos, 2–30, 31–60, and 70–207. PCA was performed using the ClustVis algorithm (https://biit.cs.ut.ee/clustvis/).

Due to their fundamental role during inflammation and viral eradication, the expression of cyto-/chemokines involved in the antiviral immune response must be quantitively fine-tuned over time. For this reason, based on the previously shown results, we performed a series of statistical analyses to find a possible association between the expression of cytokines and COVID-19 severity. According to univariate logistic regression, the odds of a severe COVID-19 diagnosis were 2.62 and 2.58 times higher in patients with a high IP-10 and TNF-α concentration at T0 compared to patients with a low concentration (*p* = 0.240 and *p* = 0.239; Table 3a,b), respectively. During convalescence, the odds of a high concentration (above the top quintile) of IP-10 and TNF-α at T1 were 11.14 and 18.14 times higher in patients with a severe COVID diagnosis compared with mild COVID (*p* = 0.046 and *p* = 0.017; Figure 3c,d). We obtained similar results in bivariate analyses when adjusting for IP-10 and TNF-α at T0 (Table 3e,f); in other terms, the predicted probability of a high concentration of IP-10 and TNF-α at T1 was higher in patients with severe compared with mild COVID also when controlled for IP-10 and TNF-α concentration at T0 (as continuous covariates, Figure 3a,b). Again, similar results were found when adjusting for time elapsed from negative test (Table 3g,h and Figure 3d): the predicted probability of a high concentration of TNF-α at T1 was higher in patients with severe compared to mild COVID, controlled for time elapsed from negative test (Figure 3e). Regarding IL-10, the odds of a low concentration (less than the second quintile) was significantly associated with COVID-19 severity (OR > 1 with *p* = 0.019; Table 3i,j); similar results were obtained by bivariate analyses (Table 3k,l; Figure 3c) and when adjusting for time elapsed from negative test (Figure 3f).

Furthermore, we performed a Principal Component Analysis (PCA) to find possible correlations among all of the deregulated cytokines (i.e., IL-10, IP-10, TNF-α, IFN-α, IL-1β, and IL-27) measured in the serum of the analyzed subjects. The analysis was conducted on the concentration values (pg/mL) of the six cytokines obtained by ELISA assay. In Figure 3g, the PCA analysis highlights a minimal variation in the first component between the positive (orange dots) and negativized groups (red, blue, and green dots), whereas a variance is observed between the positive group and the control group (purple dots). On the other hand, the distance on the PC2 axis shows a higher variance between the group of positive patients and the other groups. Collectively, the PCA highlighted how the COVID-19 disease could be a leader event that reprograms cytokine and chemokine expression, as demonstrated by reduced inter-individual variance in positive and recovered patients.

## 4. Discussion

In this study, the significance of 11 cyto-/chemokines (i.e., IL-1α, IL-1β, IL-6, IL-8, IL-10, IL-12(p70), IL-27, IFNγ, IP-10, MCP-1, TNF-α) has been analyzed in serum samples from infected and recovered subjects, possibly predictive of severity and/or duration of the disease. The serial use of wide-range analysis with a cytometric assay and with ELISA assay allowed us to find that IL-10 levels were increased in the T0 group compared to both HD and T1; IL-27 significantly decreased in the 31–60 group; IL-1β significantly increased in the 70–200 day group; TNF-α significantly decreased in T0 compared to HD and in the 31–60 day group versus HD; and IP-10 significantly increased in T0 compared to HD.

The intensity of the immunological response is linked to both the protective response and COVID-19 severity, and it is crucial for the pathophysiology of COVID-19. A variety of cytokines emerge in the orchestration of immune defense against SARS-CoV-2 infection by dictating the innate and adaptive immune system dynamics, represented by cellular and humoral response counterparts, which are relevant before, during, and after COVID-19 infection [10]. Several studies whose objective was to investigate the role of the cytokine storm in COVID-19 disease highlighted the importance of measuring the concentrations of cytokines and chemokines, correlating them to the disease progression, thereby providing preventive and more case-oriented treatments [11]. Even if the network of the molecular factors developed following a pathological event such as COVID-19 is complex and may change individually and during the disease progression, it has been reported that COVID-19 disease positively correlates with high levels of inflammatory cytokines, principally MCP-1/CCL2, IP-10, and anti-inflammatory cytokines such as IL-10 [12]. In our study, six cyto-/chemokines appeared to be deregulated among the groups of the analyzed subjects (i.e., IL-8, IL-10, IP-10, TNF-α, IL-1β, IL-27). The IL-10 increase in the serum of T0 patients observed in our hands retraced the data obtained by Korobova et al. [5]. 

Even if we did not observe an increase in IL-27 in T0 patients as in Korobova et al., this cytokine was found elevated in recovered T1 patients, again supporting a protective role for this cytokine. Indeed, it has been reported that decreased IL-27 serum levels are associated with severe COVID-19 and fatalities [13].

Compared to healthy donors, IL-8 downmodulation was also observed by Carreto-Binaghi et al., when blood cells were stimulated with NOD-like and TLR agonists. After 14 days, cells regained responsiveness to NOD-like and TLR agonists [14]. In our case, serum levels of IL-8 are even lower at T1, suggesting that even if producer cells retrieved the capacity to release IL-8 in response to pathological stimuli, homeostatic serum levels of IL-8 were reacquired with a slower kinetic. 

Regarding the upregulation of IP-10 in the sera of T0 patients, again, our results matched those of Tripathy et al., who found elevated levels of IP-10 in the sera of positive patients as well as superimposable levels in the control and recovered individuals [15]. 

Conversely, in our study we did not find any increase in both IL-1β and TNF-α in T1 convalescent patients. Indeed, these cytokines were downmodulated in T0 patients. The latter results are partially in contrast with those reported by Korobova et al. about an increase in the levels of TNF-α in patients infected by the specific alpha variant of SARS-CoV-2 [5]. Conflicting results were obtained for IL-1β serum levels, which some authors have reported to be elevated [16], whereas others observed no changes [17] or even a decrease, as we did, depending on the day of the blood withdrawal [18]. As in our case, the blood withdrawals of positive patients were carried out at admittance; our results are in line with those obtained by Yudhawati et al. [18].

To confirm the data obtained by the cytometric cytokine bead assay and to obtain more insights into the kinetics of cyto-/chemokine deregulation in the convalescent phase, we performed a set of ELISA tests focusing on the six cyto-/chemokines previously identified as altered during COVID-19 infection, also including a test for the levels of IFN-α, which was not included in the previous cytokine bead assay. We also expanded the patient’s cohort, and we classified the convalescent patients according to the time elapsed from negativization. Results previously obtained from IL-10, IP-10, and TNF-α were essentially confirmed by ELISA as well. In addition, we observed an increase in serum levels of IL-1β in the group of convalescents from 70 to 207 days, as reported by Gong et al. [19]. However, we observed a decrease in TNF-α in the group of convalescents from the 31–60 day group. 

Of note, we did not confirm by ELISA the slight IL-27 upregulation recorded by the cytometric bead assay in T1 patients. On the contrary, in one of the convalescent subgroups (31 to 60 days) IL-27 levels seemed to be downregulated. This discrepancy could be explained by the different sensitivities of the two assays. Indeed, the cytometric assay is based on fluorescence and is, therefore, a more sensitive detection method. We also measured a sizable IFN-α release in the sera of some positive patients.

In the attempt to correlate clinical course with cyto-/chemokine levels measured in the patient’s sera, we matched clinical characteristics with the observed alterations in the identified cyto-/chemokine level pattern. A direct correlation was found between IP-10 concentration and the development of severe COVID-19 (Table 3a; Figure 3a,c). This scenario is similar to some others occurring in several diseases associated with different viral infections. Elevated concentration of IP-10 has been determined in both human immunodeficient virus/human hepatitis C virus (HIV/HCV) patients compared to healthy donors as well as in the peripheral blood from patients infected with influenza A virus (H1N1) [20,21,22]. Matching the data obtained for TNF-α at T1 in severe patients (Table 3b and Figure 3b,d), these two factor levels appear to be tightly correlated. Results for IP-10 validate the possible use of sera levels of this chemokine as a predictive marker of disease severity [23,24]. 

Another striking feature is the inverse association we found between the development of severe COVID-19 and sera levels of IL-10 (Table 3i,j). Considering that this cytokine is an essential negative regulator of inflammation and immune response [25], the results we obtained are in line with the scenario of a lack of immunological response in the patients developing a severe and even fatal disease due to the settlement of the cytokine storm. In some patients, the level of IL-10 remained elevated at T1 as well as at 31–60 days, suggesting that in these patients the sequelae of COVID-19, especially from the inflammatory point of view, might be prolonged, thereby laying the foundations for the development of long COVID-19 syndrome [26]. Regrettably, we could neither have a follow up of the few patients with elevated IL-10 serum levels in the convalescent phase nor ascertain the possible presence of polymorphisms associated with lower IL-10 circulating levels as reported in individuals infected with malaria [27]. It is, again, tempting to speculate that these differences could be attributed to genetic polymorphisms in key elements of IL-10 synthesis and release and that these features could play a role in the difference between patients with or without long COVID-19 syndrome.

## 5. Conclusions

In conclusion, the analysis of cyto-/chemokine levels in a small cohort of Italian hospitalized patients confirmed and deepened our understanding of the potential role of IP-10 as a potential prognostic disease marker. Moreover, our results show that testing IL-27 and IL-10 levels may be useful in clarifying the possible onset of severe disease and long COVID-19 syndrome, respectively.

Some limitations in our study should be underlined: (i) the small number of subjects in our cohorts; (ii) the recruitment of subjects of the same ethnic group (Caucasian) and located in the province of Latina, Italy; (iii) the short period of recruitment from March 2020 to the beginning of 2022 that excludes from the analysis the last variants of SARS-CoV-2. By contrast, some strengths can be also highlighted: the flow cytometry assay has the ability to determinate a plethora of mediators at the same time; the subsequent ELISA analysis reinforces the previous type of analysis, therefore providing more complete results.

## Figures and Tables

**Table 1:** Demographic and clinical characterization of subjects analyzed by multiplex cytokine assay.

2 doses of BNT162b2 vaccinated healthy donors (HDs) (number)	10
Male (%)	10
Female (%)	90
Average age ± SD	41.6 ± 12.06
Not vaccinated patients (PZ) (number)	29
Male (%)	65.51
Female (%)	62.07
Average age ± SD (years)	60.31 ± 15.89
Time elapsed since end of infection (days) ± SD	71.86 ± 29.94
Mild (number)	15
Mild (%)	51.7
Severe (number)	14
Severe (%)	48.2

**Table 2:** Demographic and clinical characterization of subjects analyzed by ELISA.

Healthy donors (HD) (number)	25
Not vaccinated	6
3 doses of BNT162b2 vaccinated	19
Male (%)	28
Female (%)	72
Average age ± SD	47.27 ± 16.18
Patients (PZ) (number)	33
Not vaccinated	24
3 doses of BNT162b2 vaccinated	9
Male (%)	66.7
Female (%)	33.3
Average age ± SD (years)	47.48 ± 16.29
Time elapsed since end of infection (days) ± SD	63 ± 71.53
(1° group) 2–30 days (number)	14
Average time elapsed since end of infection (days)	15 ± 9.20
(2° group) 31–60 days (number)	12
Average time elapsed since end of infection (days)	39 ± 6.68
(3° group) 70–207 days (number)	7
Average time elapsed since end of infection (days)	161 ± 94.98
Mild (number)	7
Mild (%)	21.21
Severe (number)	3
Severe (%)	9.09
Pauci-symptomatic (number)	8
Pauci-symptomatic (%)	24.24
Pneumonia (number)	15
Pneumonia (%)	45.45

**Table 3:** (a) Univariate logistic model at T0. (b) Univariate logistic model at T0. (c) Univariate logistic model at T1. (d) Univariate logistic model at T1. (e) Bivariate logistic model at T1 for IP-10. (f) Bivariate logistic model at T1 for TNF-α. (g) Bivariate logistic model at T1 for IP-10. (h) Bivariate logistic model at T1 for TNF-α. (i) Logistic model at T0 for IL-10. (j) Logistic model at T1 for IL-10. (k) Bivariate logistic model at T1. (l) Bivariate logistic model at T1.

(a)				
Odds Ratio of Event = Severe COVID-19				
Variable	Estimate	95% CL		*p*-Value
IP10_T0 high vs. low *	2.62	0.53	13.10	0.240
* high: ≥the top quintile vs. low: <the top quintile (i.e., ≥873 vs. <873).				
(b)				
Odds Ratio of Event = Severe COVID-19				
Variable	Estimate	95% CL		*p*-Value
TNF-α_T0 high vs. low *	2.58	0.54	13.15	0.239
* high: ≥the top quintile vs. low: <the top quintile (i.e., ≥153 vs. <153).				
(c)				
Odds Ratio of High IP-10 Concentration at T1 *				
Variable	Estimate	95% CL		*p*-Value
severe COVID-19 vs. non-severe	11.33	1.05	122.55	0.046
* high: ≥the top quintile vs. low: <the top quintile (i.e., ≥363 vs. <363).				
(d)				
Odds Ratio of High TNF-α Concentration at T1 *				
Variable	Estimate	95% CL		*p*-Value
TNF-α high vs. low *	18.00	1.69	191.52	0.017
* high: ≥the top quintile vs. low: <the top quintile (i.e., ≥212 vs. <212).				
(e)				
Adjusted Odds Ratio of High IP-10 Concentration at T1 *				
Variable	Estimate	95% CL		*p*-Value
severe COVID-19 vs. non-severe	13.51	1.08	191.80	0.050
IP-10 at T0 **	2.62	0.77	8.84	0.120
* high: ≥top quintile vs. low: <top quintile (i.e., ≥363 vs. <363)				
** for one natural log increment				
(f)				
Adjusted Odds Ratio of High TNF-α Concentration at T1 *				
Variable	Estimate	95% CL		*p*-Value
severe COVID-19 vs. non-severe	16.52	0.83	328.37	0.066
TNF-α at T0 **	35.70	1.41	906.61	0.030
* high: ≥80th percentile vs. low: <80th perc. (i.e., ≥212 vs. <212)				
** for one natural log increment				
(g)				
Adjusted Odds Ratio of High IP-10 Concentration at T1 *				
Variable	Estimate	95% CL		*p*-Value
severe COVOD-19 vs. non-severe	11.78	1.15	120.28	0.037
months from negative test **	1.25	0.47	3.34	0.656
* high: ≥top quintile vs. low: <top quintile (i.e., ≥363 vs. <363)				
** for 3 months increments				
(h)				
Adjusted Odds Ratio of High TNF-α Concentration at T1 *				
Variable	Estimate	95% CL		*p*-Value
severe COVID-19 vs. non-severe	4.62	0.24	90.05	0.312
months from negative test **	10.90	0.50	253.71	0.128
* high: ≥80th percentile vs. low: <80th perc. (i.e., ≥212 vs. <212)				
** for 3 months increment				
(i)				
Odds Ratio of Event = Severe COVID-19				
Variable	Estimate	95% CL		*p*-Value
IL-10 low vs. high *	1.47	0.21	10.20	0.699
* low: ≤second quintile vs. high: >second quintile (i.e., ≤1.3 vs. >1.3)				
(j)				
Odds Ratio of Low IL-10 Concentration at T1 *				
Variable	Estimate	95% CL		*p*-Value
severe COVID-19 vs. non-severe	8.17	1.42	47.02	0.019
* low: ≤second quintile vs. high: >second quintile (i.e., ≤1.3 vs. >1.3)				
(k)				
Odds Ratio of Low IL-10 Concentration at T1 *				
Variable	Estimate	95% CL		*p*-Value
severe COVID-19 vs. non-severe	6.43	1.05	39.40	0.032
Il-10 at T0 **	2.86	0.47	17.54	0.256
* low: ≤second quintile vs. high: >second quintile (i.e., ≤3.9 vs. >3.9) ** for one natural log increment				
(l)				
Odds Ratio of Low IL-10 Concentration at T1 *				
Variable	Estimate	95% CL		*p*-Value
severe COVID-19 vs. non-severe	4.47	0.51	39.52	0.178
months from negative test **	2.27	0.18	29.05	0.528
* low: ≤second quintile vs. high: >second quintile (i.e., ≤3.9 vs. >3.9) ** for 3 months increment				

## Data Availability

Data is contained within the article.
